# Monocentric Prospective Study into the Sustained Effect of Incobotulinumtoxin A (XEOMIN^®^) Botulinum Toxin in Chronic Refractory Migraine

**DOI:** 10.3390/toxins10060221

**Published:** 2018-06-01

**Authors:** Ioana Ion, Dimitri Renard, Anne Le Floch, Marie De Verdal, Stephane Bouly, Anne Wacongne, Alessandro Lozza, Giovanni Castelnovo

**Affiliations:** 1 Department of Neurology, Nimes University Hospital, 30900 Nimes, France; dimitri.RENARD@chu-nimes.fr (D.R.); anne.LEFLOCH@chu-nimes.fr (A.L.F.); marie.DEVERDAL@chu-nimes.fr (M.D.V.); stephane.BOULY@chu-nimes.fr (S.B.); anne.WACONGNE@chu-nimes.fr (A.W.); giovanni.castelnovo@chu-nimes.fr (G.C.); 2 Neurological Institute, Foundation Casimiro Mondino, 27100 Pavia, Italy; alessandro.lozza@mondino.it

**Keywords:** refractory chronic migraine, tension headache, medication *overuse headache*, prophylactic treatment, XEOMIN^®^

## Abstract

Refractory chronic migraine is a disabling disorder impacting quality of life. BOTOX^®^ (Onabotulinumtoxin A) is approved as a prophylactic treatment of chronic migraine in patients unresponsive to at least three prior preventive treatments. The objective of this study was to determine the prophylactic effect of 145 U XEOMIN^®^ (Incobotulinumtoxin A) injected at 31 specific sites in adult patients with refractory chronic migraine. Sixty-one patients (8 men and 53 women, mean age 50) with migraine were recruited, including 20 patients with isolated chronic migraine, 18 patients with chronic migraine associating tension-type headache, 12 patients with migraine associating medication *overuse headache*, and 11 patients with episodic disabling migraine. The mean number of injections and duration of treatment per patient was 3.5 (range 2–13) and 21 (6–68) months, respectively. From baseline to first injection, 44 patients (73%) had >50% reduction in frequency of migraine episodes, 29 patients (48%) showed >50% reduction in number of headache days, and 28 patients (46%) had a >50% reduction in drug intake. Stable response for all three parameters was observed after the last injection. XEOMIN^®^ thus seems to represent an effective and sustained prophylactic treatment of chronic migraine.

## 1. Introduction

Between 2% and 15% of the world’s population suffers from migraines, with a wide variation of frequency of attacks [1]. Refractory chronic migraine (CM) is a disabling illness causing significant interference with quality of life, despite promising results from trials of acute and prophylactic treatments. CM affects up to a fifth of migraine patients [1].

Despite the development of new pathophysiological hypotheses (and associated recent advancements on drug development), the benefit of the majority of conventional migraine preventive drugs does not exceed 50% over placebo [2]. A recent meta-analysis shows that only high dose topiramate and sodium valproate were more effective than placebo at reducing migraine by more than 50% [3].

The National Institute for Health and Clinical Excellence (NICE) and the US Food and Drug Administration have both recently approved BOTOX^®^ (botulinum toxin type A—BoNT/A) for the prophylaxis of CM, specifically in refractory (i.e., not responding to at least three prior prophylactic treatments) CM patients [4,5]. In studies analyzing efficacy of BOTOX^®^ in migraine patients, treatment also seemed to be effective in patients with migraine and associated medication *overuse headache* [MOH]) [6]. It was recently suggested that BOTOX^®^ also represents an effective and safe intervention to target psychiatric comorbidities of migraine with improvements in depression and anxiety [7]. Double-blind, placebo-controlled trials of toxin A injections in patients with isolated episodic disabling migraine headaches (EDM) or tension-type headache (TTH) did not show significant effect of BoNT/A onabotulinum, even after controlling for a high placebo effect and after dose stratification [8,9,10]. However, patient numbers were low in these studies. Available clinical trials analyzing BONT/A efficacy in patients with isolated TTH show conflicting data, with a majority of studies showing no efficacy [11].

Botulinum toxin A efficacy has never been analyzed in patients with migraine and associated TTH.

XEOMIN^®^ inhibits the release of acetylcholine from peripheral cholinergic nerve endings acting like a myorelaxant, but also like an analgesic by suppressing the peripheral and central sensitization [12]. Unlike other neurotoxins, XEOMIN^®^ triggers minimal allergic reactions as it has no binding albumin protein [13].

To the best of our knowledge, XEOMIN**^®^** efficacy in migraine or other headache types has never been reported. The objective of our study was to assess the effect of XEOMIN^®^ treatment in refractory CM (isolated or associated with TTH or MOH) and EDM patients.

## 2. Results

Demographic and clinical features of patients are shown in Table 1. Sixty-one patients (8 men and 53 women; mean age 50, range 22–73) were recruited, including 20 patients with isolated CM, 18 with CM-TTH, 12 with CM-MOH, and 11 with EDM. Before inclusion, nonsteroidal anti-inflammatory drugs, paracetamol, morphine, and triptans were used in 18, 20, 9, and 32 of the 61 patients, respectively. The mean number of injections and mean duration of treatment per patient was 3.5 (range 2–13) and 21 (range 6–68) months, respectively (Table 2).

For the entire patient group, between baseline and the episode after the last injection, a >50% reduction in frequency of migraine episodes and headache days was observed in 44 (73%) and 29 patients (48%), respectively, whereas a >50% decrease in drug intake was observed in 28 patients (46%). In total, 44 patients (73%) were considered responders, including 18 CM, 16 CM-TTH, 6 CM-MOH, and 4 EDM patients. The 17 non-responders showed absence of therapeutic response in six patients and <50% response in 11 patients after two injections.

Compared to non-responders, responders showed >50% reduction of consumption of any acute medication after a mean of two years of treatment (*p* < 0.001). The mean number of pain-relief pills per month reduced from baseline to the episode after the last injection: 51 to 18 for morphine, 67 to 14 for paracetamol, 12 to 4 for triptan, and 12 to 5 for nonsteroidal anti-inflammatory drugs.

**Figure 1:**
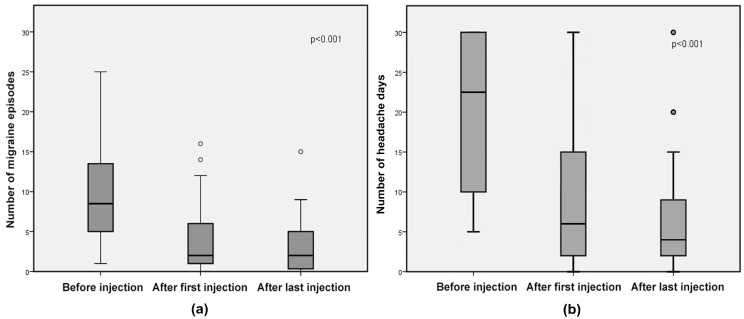
Responder group: box-and-whisker diagrams, presented as medians and interquartile ranges of (**a**) number of migraine episodes and (**b**) number of headache days. Circles represent outliers.

Median migraine episodes decreased from 8.5 (range 1–30) at baseline to 2 (range 0–16) after the first injection (*p* < 0.001) and to 2 (range 0–15) after the last injection (*p* < 0.001) (without difference in efficacy between the first and last injection, *p* = 0.3), corresponding to a 76% decrease from baseline to both later time points (Figure 1). Mean headache days decreased from 20.8 (SD 9.6) at baseline to 8.5 (SD 8.2) after the first injection (*p* < 0.001) and to 7.3 (SD 7.6) after the last injection (*p* < 0.001) (without difference in efficacy between the first and last injection, *p* = 0.4), corresponding to a 41% and 36% decrease from baseline to the first and last injection, respectively (Figure 1).

**Figure 2:**
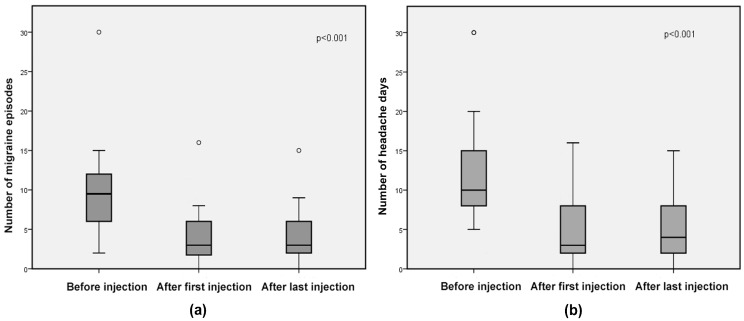
Isolated chronic migraine subgroup: box-and-whisker diagrams, presented as medians and interquartile ranges of (**a**) number of migraine episodes and (**b**) number of headache days. Circles represent outliers.

In the subgroup of patients with isolated CM (Figure 2), mean migraine episodes decreased from 9.83 (SD 6.23) at baseline to 4.06 (SD 4.04) and 4.26 (SD 3.98) after the first (59% reduction, *p* < 0.001) and last injection (57% reduction, *p* < 0.001), respectively (without difference in efficacy between first and last injection, *p* = 0.8); mean number of headache days was reduced from 13.11 (SD 7.71) at baseline to 4.40 (SD 4.27) (69% decrease, *p* < 0.001) after the first injection and to 4.66 (SD 4.21) after the last injection (67% decrease, *p* < 0.001) (without difference in efficacy between the first and last injection, *p* = 0.9).

**Figure 3:**
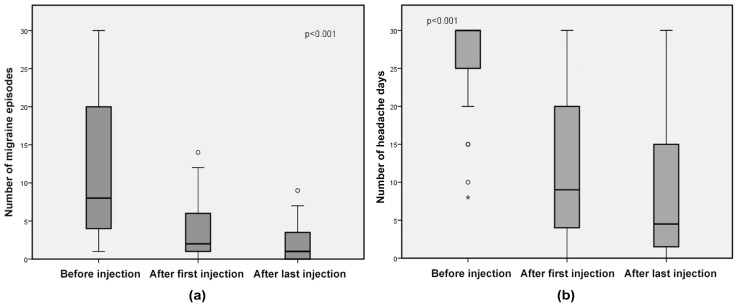
CM/TTH, CM-MOH and EDM subgroup: box-and-whisker diagrams, presented as medians and interquartile ranges of (**a**) number of migraine episodes and (**b**) number of headache days. Circles represent outliers.

In the subgroup of patients with headache type other than isolated CM (Figure 3), the median number of migraine episodes decreased from 8 (range 1–30) at baseline to 2 (range 0–14) after the first injection (69% decrease, *p* < 0.001) and to 1 (range 0–9) (93% decrease, *p* < 0.001) after the last injection (without difference in efficacy between the first and last injection, *p* = 0.1), and median number of headache days from 27 (range 8–30) at baseline to 9 (range 0–30) after the first injection (57% decrease, *p* < 0.001), and to 4.5 (0–30) after the last injection (69% decrease, *p* < 0.001) (without difference in efficacy between the first and last injection, *p* = 0.3).

When comparing both subgroups (isolated CM vs. non-isolated CM), non-isolated CM patients showed more frequent treatment response than isolated CM patients (*p* < 0.001 for both number of migraine episodes between baseline and first injection and between baseline and last injection; *p* < 0.001 for both the number of headache days between baseline and first injection and between baseline and last injection; *p* < 0.001 for both reduction in drug intake between baseline and first injection and between baseline and last injection).

The treatment was generally well tolerated. The most frequent adverse events reported were neck pain (7%) and flu-like syndrome (5%), but all these symptoms were transitory, disappearing after a maximum of 72 h, did not interfere with patient activity, and did not need further management. Overall, no patients discontinued treatment due to adverse events.

## 3. Discussion

In this study, XEOMIN^®^ proved to be an effective prophylactic treatment with sustained efficacy in patients with a refractory CM and EDM. Treatment efficacy was observed both in patients with isolated migraine and in patients with migraine and associated TTH or MOH. XEOMIN^®^ was well tolerated and no serious adverse events were observed.

To the best of our knowledge, this is the first prospective study analyzing the prophylactic efficacy of XEOMIN^®^ in patients with refractory migraine and the efficacy of botulinum toxin in patients with migraine and associated TTH.

The PREEMPT 2 study has demonstrated that BOTOX^®^ is effective for prophylaxis of adult CM patients [14]. BoNT/A treatment was also effective (significant reduction of headache days and triptan intake) in the subgroup of patients with CM and associated MOH in the PREEMT study [6].

One retrospective case series reporting on 21 Xeomin^®^-treated CM patients showed improvement in headache severity and frequency [15].

The treatment response we found in our XEOMIN^®^ study corresponded to that observed in the earlier BOTOX^®^ study [16]. However, in contrast with the PREEMPT 2 study where only the number of headache/migraine days was analyzed, we also assessed the number of migraine episodes and the reduction in drug intake. Our data support evidence that XEOMIN^®^ treatment is effective on all three headache parameters.

In our study, the incobotulinum effect was sustained (lasting from the very first injection to the last) in all responders.

Study limitations included the lack of a control group (because of the known high placebo effect for headache treatment and especially for injectable therapies), the small sample size, and the non-inclusion of specific tools assessing depression, disability and quality of life. The long-term follow-up of the patients included in our study is ongoing.

A double-blind BOTOX^®^ and XEOMIN^®^ treatment study should be conducted in more patients (and compared with a placebo arm) with isolated migraine and with migraine associating other headache types in order to compare the efficacy of both BoNT/A treatments in the different patient groups.

## 4. Conclusions

These results suggest that IncobotulinumtoxinA toxin may be an effective and safe prophylactic treatment for a variety of refractory migraines. Its effect is sustained over time, reducing medication overuse, as suggested in the open-label phase of PREEMPT.

## 5. Materials and Methods

Between August 2009 and January 2016, we invited all adult patients with CM (isolated or associated with TTH or MOH) to a specialized headache consultation at our center (Nîmes University Hospital, France) or with EDM to be included in our study. The diagnosis of the different headache types analyzed in our study fulfilled the criteria of the International Classification of Headache Disorders ICHD-3 [17]. Pregnancy was an exclusion criterion. Signed written informed consent was obtained in all included patients.

Each patient was injected with a total of 145 UI of XEOMIN^®^ at 31 specific points, topographically similar to the myogenic trigger points associated with referred pain locations (facial, pericranial and cervical muscles) [7]. The injections were scheduled at an interval between 3 and 6 months.

We prospectively evaluated the benefit of XEOMIN^®^ by calculating the number of migraine episodes and headache days, and the drug intake (expressed in number of pills of nonsteroidal anti-inflammatory drugs, paracetamol, morphine, and triptans) during the six months preceding the first injection, during the 3 to 6 months after the first injection, and during the 3 to 6 months after the last injection.

Responders were defined as patients with at least 50% reduction in frequency of migraine episodes and/or headache days. Responder analyses were performed for the entire patient group and for 2 pre-specified patient subgroups (i.e., patients with isolated CM and patients with headache type other than isolated CM). Further injections were stopped in case of non-response after 2 injections.

The software used for statistical analysis was Statistical Package for Social Sciences (SPSS 20). Paired *t*-test was used to study differences between outcome variables (with *p* < 0.05 deemed as significant difference). Median was used for the groups with abnormal distribution, and mean for the rest of the analyses when permitted.

## Figures and Tables

**Table 1:** Demographic and clinical features.

**Demographic Features**	
Sex: men/women	8/53
Age: years, mean ± SD	50 ± 10
**Headache type**	
Isolated chronic migraine	20 (33%)
Chronic migraine + tension-type headache	18 (29%)
Chronic migraine + medication *overuse headache*	12 (20%)
Episodic disabling migraine	11 (18%)

**Table 2:** Treatment effect of XEOMIN^®^.

XEOMIN^®^ responders	44 (73%)
Mean number of injections	3.5 (2–13)
Mean duration of treatment (months)	21 (6–68)
Mean duration of effect (weeks)	13.63
